# Annotations

**Published:** 1898-06-11

**Authors:** 


### ANNOTATIONS.

### Mortality in War.

The question of the possible mortality among those engaged in military operations is one of great interest just now to insurance companies in America, and efforts have been made to learn the proportion of the losses to the total number of troops in the American Civil War. It appears that the number of enlistments (reduced to a three years' basis) is estimated at 2,320,272, and that the mortality is summed up as follows: Killed and died of wounds, 110,070, or 47 per 1,000; died of disease, 224,586, or 97 per 1,000; died from accident and all other causes, 24,872, or 11 per 1,000; total, 359,528, or 155 per 1,000 for the three years' term. Thus the average death-rate per year among those enlisted works out roughly at 52 per 1,000. Of course, in any attempt at a comparison of this death-rate with that of the civil population, it must be borne in mind how low is the normal death-rate between the ages of 20 and 35, the age period into which the great mass of the soldiers would fall. It is also worthy of note how large a proportion of the mortality was due to disease. War, pestilence, and famine proverbially go together; but, quite irrespective of what one may call pestilence, real warfare produces much disease which shortens the lives even of the strongest.

### The After-care of the Insane.

There is a modest charity which does a most useful work, but which is little known to the public. This is the "After-care Association for Poor Persons Discharged Recovered from Asylums for the Insane." Mental convalescents are much like those recovering from physical illness; they may be too well to be kept in a hospital, and yet not well enough at once to take up the battle of life. In almost all cases, we should think, it would be advisable that a lunatic who has recovered his sanity in an asylum should be kept under some kind of supervision for a time, in order to make sure that under the conditions of ordinary life he will still keep his reason. He cannot be trusted all at once to take up his former interests and anxieties without their effect upon his intellect being watched. For this reason something of the nature of a convalescent home for asylum patients is desirable. Perhaps even better is it to board these convalescents in a family for a few weeks, where they can fall at once into the ways of ordinary life, free from the restrictions of an institution, and yet carefully and kindly watched, lest any sign of irregular intellect should betray itself. Even when the former lunatic is fully recovered and is fit for work, he often finds it difficult to obtain it. His record makes people chary of employing him, and anxiety and depression at his inability to find employment may, and frequently does, plunge him back into his old condition. It is in such cases that this association, the only one of the kind in the country, steps in. During the past year it has dealt with the cases of 147 recovered lunatics. It has not expended its funds upon those whom, after due consideration, the council of the association has come to the conclusion would never be fit for the struggle of life again; but those whom they thought could be effectually helped have been assisted in various ways. Some have been boarded in cottage homes in the country where the conditions of life were healthy and not too exciting, and where they could be, so to speak, broken into ordinary ways. Others have received grants of money or clothing; and others again have been assisted in finding work. It is probable that some, at least, of these would, if no such help had been given, have relapsed into madness again. The value of the work cannot be denied, and in its loving care for these unfortunate sufferers it is most Christ-like. Unfortunately, it is, as we have said, not well-known, and its resources are small. Its total income last year was £561 7s. 6d. As many of the cases require the most careful personal inquiry on the part of the secretary or some other trustworthy person, the expense of distribution cannot but be rather large; but, on the other hand, there is the less risk of misappropriation of funds to unsuitable or undeserving cases. If the income of the society were larger much more could be accomplished, and it is to be hoped that the appeal it makes for increased subscriptions will not fail of effect.

### A "Hard Case."

Here is a question set by the *St. James's Gazette*:—

"Among a number of officers invalided from the front who recently arrived in England viâ Marseilles was one who had only just recovered from a serious operation, one leg having been amputated high up above the knee. On being wounded he was brought into camp, and before long the surgeon attached to his regiment was investigating the injury. The pain of this process, with the loss of blood he had already experienced, caused the officer to become unconscious; but before reaching that condition he implored the surgeon on no account to amputate his leg without his knowledge and consent. To this, on the urgent request of the wounded officer, the surgeon pledged his word. A consultation being held, it was decided unanimously by the assembled surgeons that immediate amputation below the knee was indicated; every hour's delay endangered the life of the patient. Mindful of his promise, the regimental surgeon refused to allow the operation to be begun until the officer regained consciousness. When he recovered sufficiently to understand the situation and give his consent, the disease had spread so far that it was found necessary to remove the limb half-way up the thigh. In the circumstances, did the regimental surgeon act rightly in keeping his promise?"

We think he acted rightly. There can be no doubt that he was bound by his promise, and, moreover, he could not know with certainty that the delay would so seriously aggravate the condition. The propriety of making any promise of the sort is, however, another matter. Cases are constantly occurring in which surgeons land themselves in the midst of infinite embarrassment by tying themselves down beforehand as to what they will and what they will not do; and, if a surgeon is wise, he will not only refuse to operate unless he is allowed a free hand to do his best for his patient, but, in civil practice at any rate, he will require that such permission shall be given in writing. How far a soldier in enlisting, or an officer in accepting his commission gives an implied consent to submit to the medical and surgical treatment supplied by the authorities, is a question on which we will not venture to express an opinion; but if discipline does really demand that a soldier shall have his leg off at the command of or on the opinion of the medical officers who may happen to be set over him for the moment, according to the exigencies of an undermanned department, we can but feel more than ever surprised that the military element, from among whom the prospect tive patients come, should ever have allowed the medical element in that mixture of departments which we call the army to run down to its present condition.

